# Characterization and the comprehensive expression analysis of tobacco valine-glutamine genes in response to trichomes development and stress tolerance

**DOI:** 10.1186/s40529-023-00376-x

**Published:** 2023-07-10

**Authors:** Xiaoxiao Yan, Rui Luo, Xiangyang Liu, Zihang Hou, Wenyi Pei, Wenqi Zhu, Hong Cui

**Affiliations:** 1 National Tobacco Cultivation and Physiology and Biochemistry Research Center, Key Laboratory for Tobacco Cultivation of Tobacco Industry, Zhengzhou, 450002 China; 2 College of Tobacco Science, Henan Agricultural University, 63 Nongye Road, Jinshui District, Zhengzhou, China

**Keywords:** Valine-glutamine gene (*NtVQ*), Mixed-trichome (mT), Glandular-trichome (gT), Nonglandular-trichome (nT), Stress tolerance, *Nicotiana tobacum*

## Abstract

Valine-glutamine genes (VQ) acted as transcription regulators and played the important roles in plant growth and development, and stress tolerance through interacting with transcription factors and other co-regulators. In this study, sixty-one *VQ* genes containing the FxxxVQxxTG motif were identified and updated in the *Nicotiana tobacum* genome. Phylogenetic analysis indicated that *NtVQ* genes were divided into seven groups and genes of each group had highly conserved exon-intron structure. Expression patterns analysis firstly showed that *NtVQ* genes expressed individually in different tobacco tissues including mixed-trichome (mT), glandular-trichome (gT), and nonglandular-trichome (nT), and the expression levels were also distinguishing in response to methyl jasmonate (MeJA), salicylic acid (SA), gibberellic acid (GA), ethylene (ETH), high salinity and PEG stresses. Besides, only *NtVQ17* of its gene family was verified to have acquired autoactivating activity. This work will not only lead a foundation on revealing the functions of *NtVQ* genes in tobacco trichomes but also provided references to *VQ* genes related stress tolerance research in more crops.

**Supplementary Information:**

The online version contains supplementary material available at 10.1186/s40529-023-00376-x.

## Introduction

Valine-glutamine genes (VQ) were kinds of transcription regulators and played the important roles in plant growth, development and responses to various environmental stimulus. VQ proteins were labeled with a conserved single short FxxxVQxxTG amino acid sequence motif (pfam 05678), which was essential between protein interaction with transcript factors like WRKY (Cheng et al. 2012), and dispensable in the interaction with other genes like *MAPKs* (Pecher et al. 2014). Multiple *VQ* gene families from plant species have been identified and studied. Taking monocotyledons for examples, there were forty *VQ* genes in *Oryza sativa* (Kim et al. 2013; Jiang et al. 2018), sixty-one in *Zea mays* (Song et al. 2016), and eighteen in *Vitis vinifera* (Wang et al. 2015); taking dicotyledons for another examples, there were thirty-four *VQ* genes in *Arabidopsis thaliana* (Cheng et al. 2012), twenty-six in *Solanum lycopersicum* (Ding et al. 2019), and eighty-nine in *Gossypium hirsutum* (Chen et al. 2020b).

*VQ* genes were involved in multiple regulatory aspects of plant growth and development. For instance, *AtVQ8* mutants showed pale-green and stunted-growth, which was similar with the roles of *AtVQ17*/*18*/*22* in overexpression *Arabidopsis* plants (Cheng et al. 2012). *AtVQ14* (HAIKU1, *IKU1*) regulated endosperm growth and seeds size by interacting with *AtWRKY10* (Wang et al. 2010). *AtVQ20* participated in pollen development via inhibiting the expression of downstream *MYBs* genes (Lei et al. 2017). *AtVQ29* acted as a repressor in the light-meditated inhibition of hypocotyl elongation during early seedling development (Cheng et al. 2012). Moreover, *OsVQ13* could positively regulate grain size in rice (Uji et al. 2019). Soybean *VQ* genes overexpression plants showed altered leaf morphology and flowering time (Zhou et al. 2016).

*VQ* genes were also verified to manage responses to plant biotic and abiotic stress. In *Arabidopsis*, both *AtVQ4* (MPK3/6-targeted VQ protein1, MVQ1) and *AtVQ21* (Mitogen-activated Protein Kinnase4 Substrate1, *MKS1*) met with the quick response to *WRKY* mediated immune defense (Andreasson et al. 2005; Pecher et al. 2014). *AtVQ5*/*20*, *AtVQ16* (Sigma Factor-Interacting Protein2, *SIB2*) and *AtVQ23* (*SIB1*) were proved to be involved in the resistance to *Botrytis cinerea* (Lai et al. 2011; Cheng et al. 2012). *AtVQ9* and *AtVQ15* (*Arabidopsis* CaM-binding protein, *AtCaMBP25*) were demonstrated to regulate salinity and osmotic stresses, responsively (Hu et al. 2013b; Perruc et al. 2004). *AtVQ22* (Jasmonate-associated VQ motif gene1, *JAV1*) negatively defined the transcriptional activity of *WRKY28*/*51* responsive to injury (Hu et al. 2013a; Yan et al. 2018). Moreover, *OsVQ13*/*14*/*32* overexpression plants increased resistance to rice bacterial blight (Uji et al. 2019; Li et al. 2021). *BnVQ7* from *Brassica napus*, a *MKS1* homologous gene, enhanced disease resistance to *Leptosphaeria maculans* (Zou et al. 2021). The function of soybean *GmVQ58* were similar with *AtVQ4* and *AtVQ21* to participate in *WRKY* meditated immune defense responses (Li et al. 2020). These results indicated that most *VQ* genes were efficiently responsive to environmental conditions (Cheng et al. 2012).

As the epidermal outgrowths, trichomes have been divided into glandular- (gT) and nonglandular- (nT) type according to the secretory ability. Glandular-trichome were the site of biosynthesis and storage of large quantities of specialized metabolites (Chalvin et al. 2020), and played essential roles in the defense against biotic and abiotic factors such as pathogens attack and osmotic stress (Schuurink and Tissier 2020). However, little studies about *VQ* genes have focused on the trichomes development.

As one worldwide cultivated industrial crop, tobacco leaves were the main source of economic value surrounding with high density of trichomes. Tobacco glandular-trichome were related to various responses to salinity and heavy metal stresses (Yan et al. 2021; Zhang et al. 2021a, b), and the industrial quality of flue-cured leaves (Li et al. 2017). In this study, the information of tobacco *NtVQ* gene family were collected and updated for the performance of bioinformatics analysis. Then, the wild flue-cured tobacco K326 covered with mixed trichomes (mT) contained gT and nT, Tobacco Introduction 1112 (T.I.1112) characterized only by nT and T.I.1068 only shown gT were used to manifest the *NtVQ* genes comprehensive expression patterns, thus further indicating the importance of *NtVQ* in trichomes formation and development process. Meanwhile, several hormone treatments and abiotic stresses were conducted to evaluate *NtVQ* genes values in order to demonstrate the broad spectrum resistance functions. After the above analysis, the representative *NtVQ* genes were tested for autoactivation activity to facilitating a complete research system. This work would lay a solid theoretical foundation for exploring the crucial functions of *VQ* genes in more plants.

## Materials and methods

### Plant material and growth conditions

K326 was stored in the lab, T.I.1112 and T.I.1068 were provided from Oxford Tobacco Research Station (Oxford, North Carolina, USA). K326, T.I.1112 and T.I.1068 seedlings were grown at 25℃, 70–80% proportional humidity under 12 h / 12 h day / night cycles. For all experiments, four-week old seedlings were used. All experiments were repeated in triplicate.

### Identification of tobacco *VQ* genes

The VQ proteins of *Arabidopsis* were obtained from TAIR database (http://www.arabidopsis.org/). A Hidden Markov Model (HMM) with VQ motif was extracted from Pfam database (http://pfam.sanger.ac.uk/) (Finn et al. 2010; Guo et al. 2014) to identify all putative *NtVQ* genes from tobacco genome database (https://solgenomics.net/) and NCBI (https://www.ncbi.nlm.nih.gov/). The identified VQ proteins were determined using the SMART server (http://smart.embl.de/), and annotated based on their phylogenetic relationships (Zhang et al. 2019, Zhang et al. 2021a, b).

### Bioinformatics analysis of tobacco VQ genes

A phylogenetic tree of NtVQ proteins was constructed with MEGA 7.0 software using the neighbor-joining method (Kumar et al. 2016). The exon-intron structures were analyzed with their coding regions and full-length sequences and generated using the Gene Structure Display SERVER 2.0 (http://gsds.cbi.pku.edu.cn/). The NtVQ protein structures were determined using SMART server. *NtVQ* genes duplications were identified as previously described (Yan et al. 2017). The syntenic blocks were used to construct a synteny analysis map of the *NtVQ* genes from the Plant Genome Duplication Database (Tang et al. 2008). Diagrams were generated using Circos version 0.63 (http://circos.ca/) (Guo et al. 2014).

### Acquisition of tobacco tissues and trichomes phenotyping

Roots, stem, leaves (with its epidermis) and flowers from K326 were collected for tissue-specific expression analysis. Trichomes from K326, T.I.1112 and T.I.1068 leaves were removed using freeze-thawing method with liquid nitrogen (Yan et al. 2021). After staining by 2% Rhodamine B for 30 min, 1 cm wide leaf filaments without veins were cut and observed using the depth-of-field digital microscope (VHX-2000; KEYENCE, Osaka, Japan).

### Phytohormone treatments and abiotic stress

For exogenous phytohormone treatment, nine four-week old seedlings were sprayed with 150 µM methyl jasmonate (MeJA), 2.0 mM salicylic acid (SA), 150 µM gibberellic acid (GA), and 100 µM ethylene (ETH), respectively. Controls were cultured without any treatment. Samples were collected at 0, 1, 3, 6, and 12 h after phytohormone treatments and stored at -80℃. For abiotic stress, nine four-week old seedlings were separately treated with 300 mM NaCl and PEG-6000 (-0.5 MPa) solutions. Samples were collected at 0, 6, 24, 48, and 72 h after stress treatments and stored at -80℃.

### Transcriptional activity assay

The full-length coding sequences (CDS) of *NtVQ* genes were cloned using the tobacco genome as the template and the relative primers in Table S1. CDS of *NtVQ* genes were fused into the pGBKT7 vector, and the empty pGBKT7 vector was used as the control. All constructions were transformed into the Y2H Gold yeast strain and selected on SD/-Trp/X (X: X-α-gal) and SD/-Trp/X/A medium (A: AbA), respectively (Yan et al. 2021).

### RNA extraction and sqRT-PCR analysis

Tobacco RNA was extracted using the Total RNA Extraction Kit (R6827-01, Omega Bio-tek, USA). First-strand cDNAs were synthesized using a PrimeScript 1st Strand cDNA Synthesis Kit (TaKaRa Biotechnology, Dalian, China). *NtVQ* gene-specific primers were designed using Primer Premier 5.0 and listed in Table S1. *NtL25* was used as the reference gene. The semi-quantitative RT-PCR reactions profiles and methods were described in previous studies (Yan et al. 2017, 2021). The data were analyzed with 2^−ΔΔCt^ method, quantified using the Gene Tools software, and visualized into heat maps with TBtools software (Guo et al. 2014; Chen et al. 2020a).

### Data statistics

Data were presented using Microsoft Excel and Sigma Plot 10.0. One-way ANOVA analysis was performed using the SPSS Statistics 20.0 software (IBM China Company Ltd. Beijing, China) to assess significant differences.

## Results

### Identification and synteny analysis of *VQ* genes families

Sixty-one candidate *NtVQ* genes were identified in the tobacco genome sequence. The coding length of sixty-one *NtVQ* gene sequences ranged from 216 to 1404 bp (Table 1). The exon numbers of *NtVQ* genes ranged from one to nine, and 77.05% of these genes had one exon. The isoelectric points of thirty NtVQ proteins were alkaline and these of the remaining proteins were acidic, which indicated that *NtVQ* gene family had nearly harmonious relationship between alkaline and acidic amino acids.

**Fig. 1:**
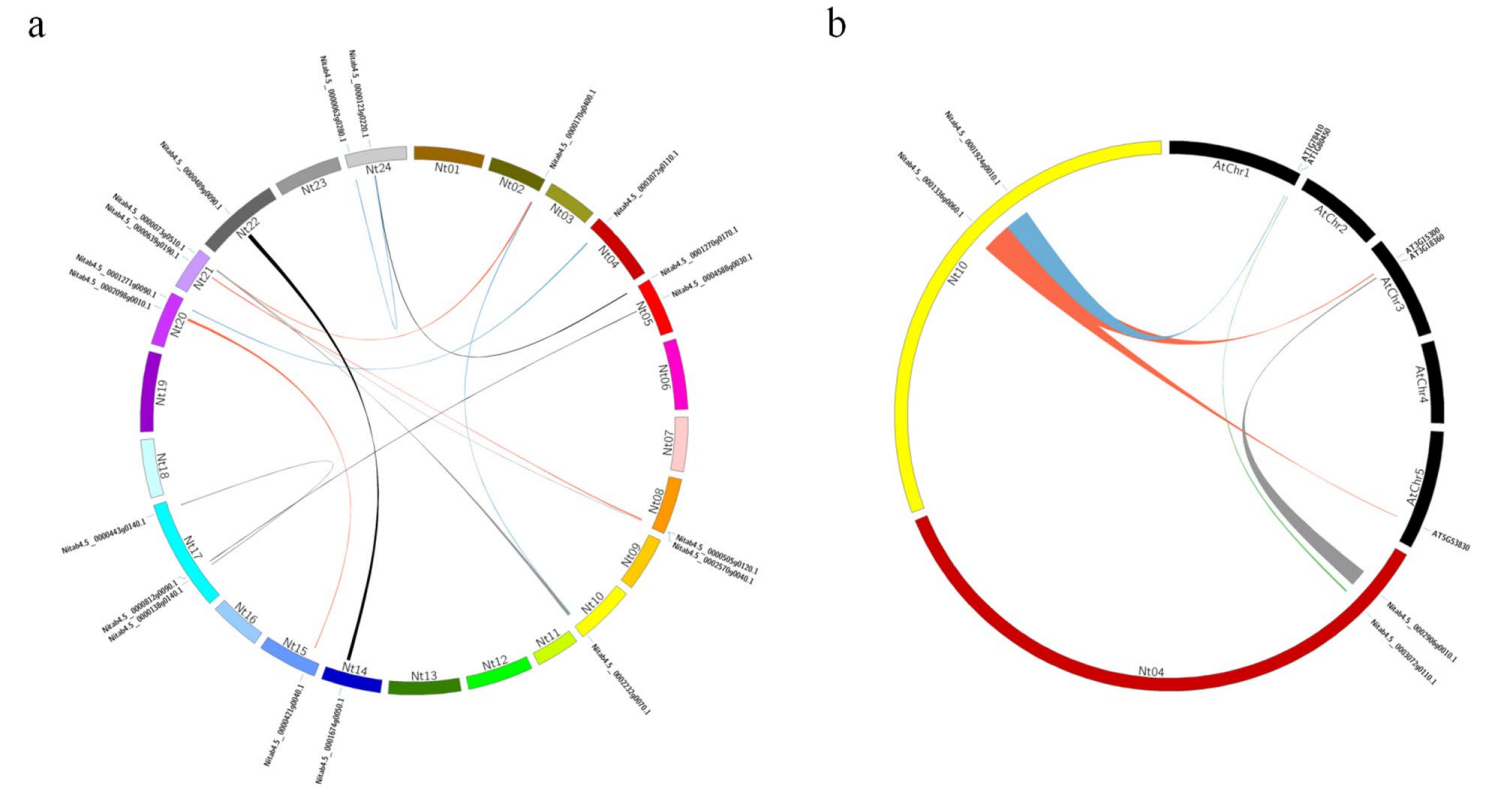
Chromosome distribution and synteny analysis of tobacco and *Arabidopsis thaliana VQ* genes (a) Chromosomes 1–24 were shown in different colors in a circular diagram. Colored curves denoted the details of syntenic regions between tobacco *VQ* genes. (b) The chromosomes of tobacco and *A*. *thaliana* were depicted as a circle. Colored curves denoted the details of syntenic regions between tobacco and *A*. *thaliana VQ* genes

As shown in Fig. 1a; Table 2, a total of nineteen *NtVQ* genes were clustered into twelve tandem duplication event regions on tobacco chromosome 2, 4, 5, 8, 10, 14, 15, 17, 20, 21, 22 and 24, indicating that less than half of the *NtVQ* genes were generated by tandem duplication. A synteny analysis from tobacco and *Arabidopsis* further showed five syntenic relations that contained four *NtVQ* genes and five *AtVQ* genes (Fig. 1b; Table 3).

**Table 1:** Tobacco *VQ* genes and accession numbers. CDS: coding sequence

Gene Locus	Gene Length	Start	End	Exon Number	CDS (bp)	Protein (aa)	Amino acid proportion (%)			pI
							Alpha helix (Hh)	Beta-sheet (E)	Loop/Random coil (L/C)	
Nitab4.5_0002305g0070.1	588	399,194	399,781	1	588	195	28/14.36%	33/16.92%	134/68.72%	5.83
Nitab4.5_0000138g0140.1	564	1,093,775	1,094,338	1	564	187	29/15.51%	28/14.97%	130/69.52%	5.92
Nitab4.5_0000443g0160.1	627	826,332	826,958	1	627	208	27/12.98%	36/17.31%	145/69.71%	4.98
Nitab4.5_0008965g0010.1	648	78,700	79,347	1	648	215	26/12.09%	45/20.93	144/66.98%	6.29
Nitab4.5_0008708g0020.1	675	84,655	85,329	1	675	224	54/24.11%	43/19.20%	127/56.70%	4.72
Nitab4.5_0000443g0140.1	675	852,301	852,975	1	675	224	60/26.79%	43/19.20%	121/54.02%	4.97
Nitab4.5_0002570g0040.1	603	93,406	94,008	1	603	200	34/17.00%	28/14.50%	137/68.50%	10.6
Nitab4.5_0000073g0510.1	600	1,448,350	1,448,949	1	600	199	27/13.57%	30/15.08%	142/71.36%	8.34
Nitab4.5_0000170g0400.1	744	926,895	927,638	2	529	202	31/15.35%	32/15.84%	139/68.81%	10.46
Nitab4.5_0002232g0070.1	612	396,497	397,108	1	612	203	25/12.32%	31/15.27%	147/72.41%	10.32
Nitab4.5_0005431g0010.1	1203	72,449	73,651	1	1203	400	68/17.00%	37/9.25%	295/73.75%	6.45
Nitab4.5_0000421g0040.1	894	164,669	165,562	1	894	297	31/10.44%	25/8.42%	241/81.14%	6.22
Nitab4.5_0002098g0010.1	879	132,258	133,136	1	879	292	24/8.22%	31/10.62%	237/81.16%	5.95
Nitab4.5_0008140g0010.1	1674	39,111	40,784	3	1338	445	43/9.66%	25/5.62%	377/84.72%	7.33
Nitab4.5_0001729g0010.1	1404	94,292	95,695	1	1404	467	32/6.85%	44/9.42%	391/83.73%	6.49
Nitab4.5_0001270g0170.1	660	438,569	439,228	1	660	219	49/22.37%	31/14.16%	139/63.47%	5.91
Nitab4.5_0000123g0220.1	660	409,929	410,588	1	660	220	45/20.55%	21/9.59%	15./69.86%	6.3
Nitab4.5_0000062g0280.1	657	1,034,829	1,035,485	1	657	218	35/16.06%	19/8.72%	164/75.23%	9.16
Nitab4.5_0010768g0030.1	10,278	14,633	24,910	9	1347	449	144/32.07%	90/20.04%	215/47.88%	5.57
Nitab4.5_0016059g0010.1	593	9637	10,229	2	321	106	21/19.81%	11/10.38%	74/69.81%	9.74
Nitab4.5_0008385g0010.1	863	402,586	41,148	2	645	214	33/15.42%	24/11.21%	157/73.36%	8.34
Nitab4.5_0000680g0150.1	516	696,743	697,258	1	516	171	30/17.54%	18/10.53%	123/71.93%	6.56
Nitab4.5_0000680g0130.1	7468	604,651	612,118	2	1167	388	84/21.65%	36/9.28%	268/69.07%	9.08
Nitab4.5_0008385g0020.1	9020	59,372	68,391	2	1143	380	98/25.79%	26/6.84%	256/67.37%	9.28
Nitab4.5_0008558g0010.1	2332	7920	10,251	3	969	322	93/28.88%	40/12.42%	189/58.70%	10.24
Nitab4.5_0000786g0140.1	875	3,998,189	399,045	2	804	267	89/33.33%	35/13.11%	143/53.56%	10.46
Nitab4.5_0004604g0010.1	948	94,021	94,968	1	948	315	21/6.67%	14/4.44%	280/88.89%	10.67
Nitab4.5_0000256g0320.1	948	966,518	967,465	1	948	315	22/6.98%	16/5.08%	277/87.94%	10.67
Nitab4.5_0003157g0050.1	558	13,932	139,880	1	558	185	26/14.05%	22/11.89%	137/74.05%	9.51
Nitab4.5_0001271g0090.1	537	577,237	577,773	1	537	178	30/16.85%	23/12.92%	125/70.22%	9.79
Nitab4.5_0003072g0110.1	567	198,841	199,407	1	567	188	22/11.70%	29/15.43%	137/72.87%	9.99
Nitab4.5_0004013g0040.1	567	191,866	192,432	1	567	188	24/12.77%	23/12.23%	141/75.00%	9.9
Nitab4.5_0001652g0050.1	783	103,509	104,291	1	783	260	28/10.77%	26/10.00%	206/79.23%	9.42
Nitab4.5_0009099g0040.1	783	73,650	74,432	1	783	360	30/11.54%	21/8.08%	203/78.00%	9.45
Nitab4.5_0001241g0040.1	687	263,071	263,757	1	687	228	32/14.04%	23/10.09%	173/75.88%	9.77
Nitab4.5_0001336g0060.1	684	557,974	558,657	1	684	227	32/14.10%	26/11.45%	169/74.45%	9.77
Nitab4.5_0003481g0110.1	453	209,282	209,734	1	453	150	58/38.67%	16/10.67%	76/50.67%	6.31
Nitab4.5_0005808g0010.1	474	8127	8600	1	474	157	54/34.39%	21/13.38%	82/52.23%	5.41
Nitab4.5_0000812g0090.1	426	586,770	587,195	1	426	141	31/21.99%	17/12.06%	93/65.96%	5
Nitab4.5_0004588g0030.1	474	228,189	228,662	1	474	157	44/28.03%	31/19.75%	82/52.23%	5.96
Nitab4.5_0000349g0050.1	10,775	152,011	162,785	2	216	71	39/54.93%	7/9.86%	25/35.21%	8.85
Nitab4.5_0002640g0020.1	1582	61,688	63,269	2	294	97	49/50.52%	15/15.46%	33/34.02%	9.94
Nitab4.5_0000687g0100.1	345	345,785	346,129	1	345	114	33/28.95%	15/13.61%	66/57.89%	6.59
Nitab4.5_0008548g0020.1	351	19,094	19,444	1	351	116	33/28.45%	15/12.93%	68/58.62%	5.87
Nitab4.5_0000930g0040.1	309	607,049	607,357	1	309	102	29/28.43%	14/13.73%	59/57.84%	5.47
Nitab4.5_0022515g0010.1	309	2522	2830	1	309	102	23/22.55%	10/9.80%	69/67.65%	6.58
Nitab4.5_0000930g0050.1	309	662,450	662,758	1	309	102	31/30.39%	9/8.82%	62/60.78%	6.25
Nitab4.5_0009599g0020.1	318	77,300	77,617	1	318	105	29/27.62%	12/11.43%	64/60.95%	5.4
Nitab4.5_0000639g0190.1	306	579,499	579,804	1	306	101	36/35.64%	15/14.85%	50/49.50%	9.63
Nitab4.5_0000505g0120.1	297	773,483	773,779	1	297	98	37/37.76%	15/15.31%	46/46.94%	9.17
Nitab4.5_0005080g0020.1	360	140,722	141,081	1	360	119	45/37.82%	15/12.61%	59/49.58%	5.34
Nitab4.5_0001924g0010.1	375	39,051	39,425	1	375	124	51/41.13%	12/9.68%	61/49.19%	5.79
Nitab4.5_0002906g0010.1	471	82,252	82,722	1	471	156	14/8.97%	26/16.67%	116/74.36%	5.45
Nitab4.5_0000489g0090.1	552	590,481	590,132	1	552	183	24/13.11%	23/12.57%	136/74.32%	5.7
Nitab4.5_0001674g0050.1	417	331,009	331,425	1	417	138	14/10.14%	23/16.67%	101/73.19%	9.17
Nitab4.5_0006679g0010.1	1554	144,320	145,873	2	455	184	28/20.65%	24/13.64%	122/66.30%	9.3
Nitab4.5_0000633g0120.1	702	299,447	300,148	2	510	169	38/22.49%	14/8.28%	117/69.23%	9.41
Nitab4.5_0000299g0270.1	765	884,677	885,441	1	765	254	58/22.83%	26/9.45%	172/67.72%	7.83
Nitab4.5_0000427g0090.1	753	785,763	786,515	2	506	167	38/22.75%	15/8.98%	114/68.26%	9.41
Nitab4.5_0004813g0010.1	678	31,487	32,164	1	678	225	43/19.11%	25/11.11%	157/69.78%	5.89
Nitab4.5_0002121g0030.1	678	283,134	283,811	1	678	225	38/16.89%	25/11.11%	162/72.00%	5.89

**Table 2:** The details of syntenic regions of tobacco *VQ* genes

Number	Gene of *Nicotiana tobacum*	Block 1			Gene of *Nicotiana tobacum*	Block 2		
		Chr	Start	End		Chr	Start	End
1	Nitab4.5_0002098g0010.1	20	68,708,367	73,291,850	Nitab4.5_0000421g0040.1	15	20,669,917	21,423,296
2	Nitab4.5_0000639g0190.1	8	99,547,870	101,524,099	Nitab4.5_0000505g0120.1	21	52,580,931	54,121,620
3	Nitab4.5_0000073g0510.1	21	70,839,788	71,923,681	Nitab4.5_0000170g0400.1	2	104,854,013	107,232,470
4	Nitab4.5_0000073g0510.1	21	71,824,456	72,399,223	Nitab4.5_0002570g0040.1	8	103,491,312	103,894,013
5	Nitab4.5_0000073g0510.1	21	70,130,728	72,003,347	Nitab4.5_0002232g0070.1	10	107,561,581	113,593,166
6	Nitab4.5_0000138g0140.1	17	57,874,512	58,956,217	Nitab4.5_0000443g0140.1	17	199,091,884	200,293,163
7	Nitab4.5_0001270g0170.1	5	138,747	1,534,446	Nitab4.5_0000123g0220.1	24	50,020,237	50,809,798
8	Nitab4.5_0000812g0090.1	17	66,442,094	66,154,875	Nitab4.5_0004588g0030.1	5	43,332,771	43,890,275
9	Nitab4.5_0001674g0050.1	22	70,052,300	78,283,777	Nitab4.5_0000489g0090.1	14	73,104,160	79,702,893
10	Nitab4.5_0000170g0400.1	2	107,224,367	108,805,080	Nitab4.5_0002232g0070.1	10	106,939,084	107,784,401
11	Nitab4.5_0000062g0280.1	24	8,997,624	9,896,916	Nitab4.5_0000123g0220.1	24	48,998,617	50,487,060
12	Nitab4.5_0003072g0110.1	4	15,579,277	17,796,517	Nitab4.5_0001271g0090.1	20	92,064,219	93,658,586

**Table 3:** The details of syntenic regions between tobacco and *Arabidopsis thaliana VQ* genes

Number	Gene of *Nicotiana tobacum*	Block 1			Gene of *Arabidopsis thaliana*	Block 2		
		Chr	Start	End		Chr	Start	End
1	Nitab4.5_0001336g0060.1	10	67,145,787	75,458,550	AT3G15300	3	5,025,184	5,196,591
2	Nitab4.5_0001336g0060.1	10	67,187,464	71,464,670	AT5G53830	5	21,822,323	21,914,866
3	Nitab4.5_0002906g0010.1	4	8,900,255	13,115,923	AT3G18360	3	6,154,363	6,399,337
4	Nitab4.5_0003072g0110.1	4	15,178,772	15,579,843	AT1G80450	1	30,243,430	30,280,359
5	Nitab4.5_0001924g0010.1	10	74,607,412	80,668,737	AT1G78410	1	29,540,984	29,468,394

### Phylogenetic analysis of *VQ* gene families

**Fig. 2:**
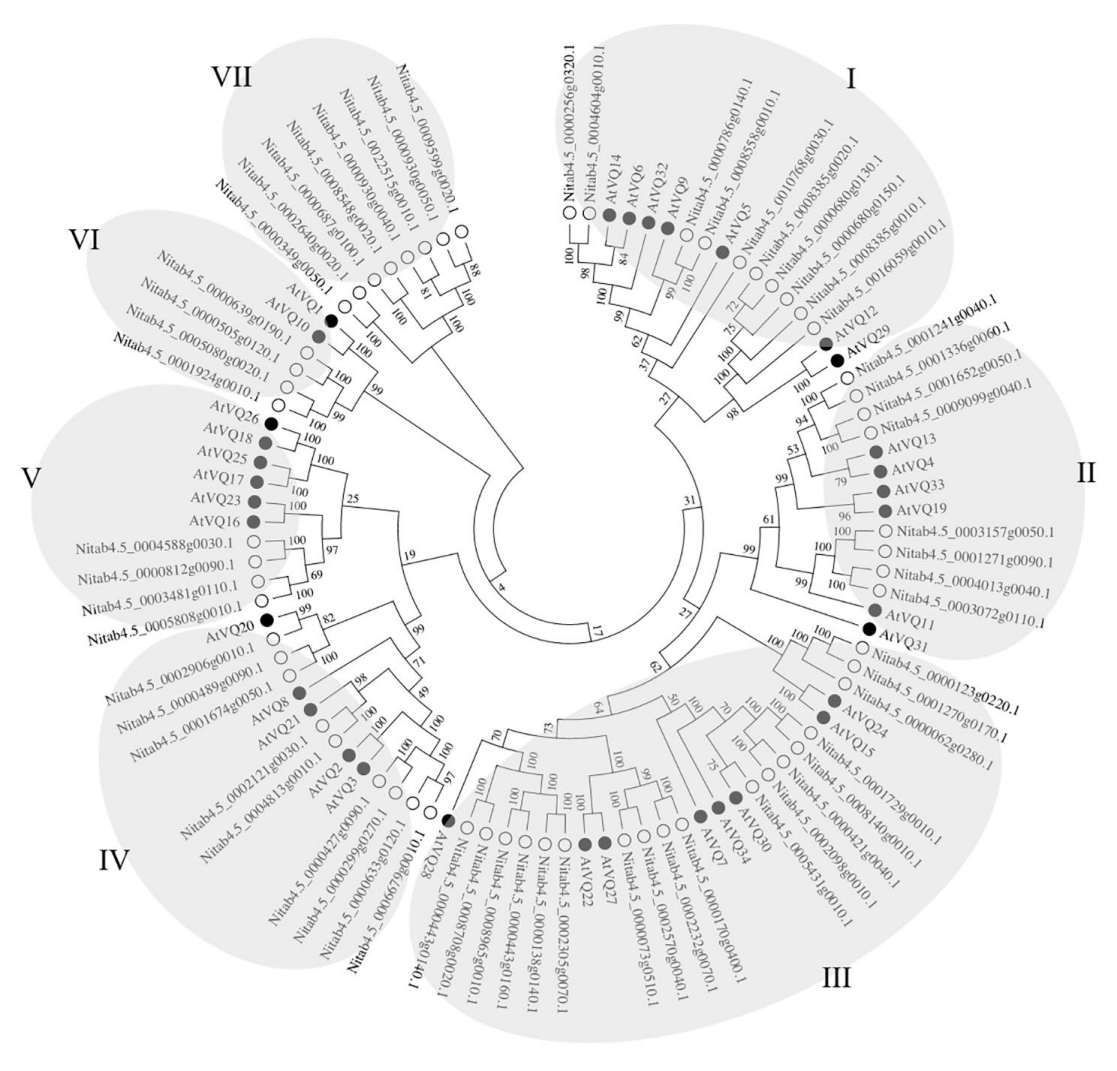
Phylogenetic analysis of VQ proteins among tobacco and *Arabidopsis thaliana* The full-length amino acid sequences of sixty-one tobacco *VQ* genes and thirty-four *Arabidopsis VQ* genes were aligned by using ClustalX and the phylogenetic tree was constructed using MEGA 7.0 by the neighbor-joining method with 1000 bootstrap. λ: *N. tabacum*, ϒ: *A. thaliana*

According to the phylogenetic tree (Fig. 2), ninety-five VQ proteins derived from *N. tobacum* and *A. thaliana* were clustered into seven groups named from I to VII. Group I - VI contained VQ proteins from both tobacco and *Arabidopsis*, and group VII contained only tobacco VQ proteins. Group III (29.50%) contained the largest number of genes, followed by group I (16.39%), group IV (14.75%), group II (13.11%), group VII (13.11%), group V (6.56%) and group VI (6.56%). Moreover, a subset of the *Arabidopsis* VQ proteins phosphorylated by the MPK3a and MPK6 (named as MVQs) were uniformly clustered into the group I and II (Pecher et al. 2014), which predicated that eighteen tobacco VQ proteins from group I and II may be involved in the cellular process of protein phosphorylation. This comparison between VQ proteins clarified that genes in the same group may have similar functions.

### Structure and phylogenetic analysis of *NtVQ* gene family

**Fig. 3:**
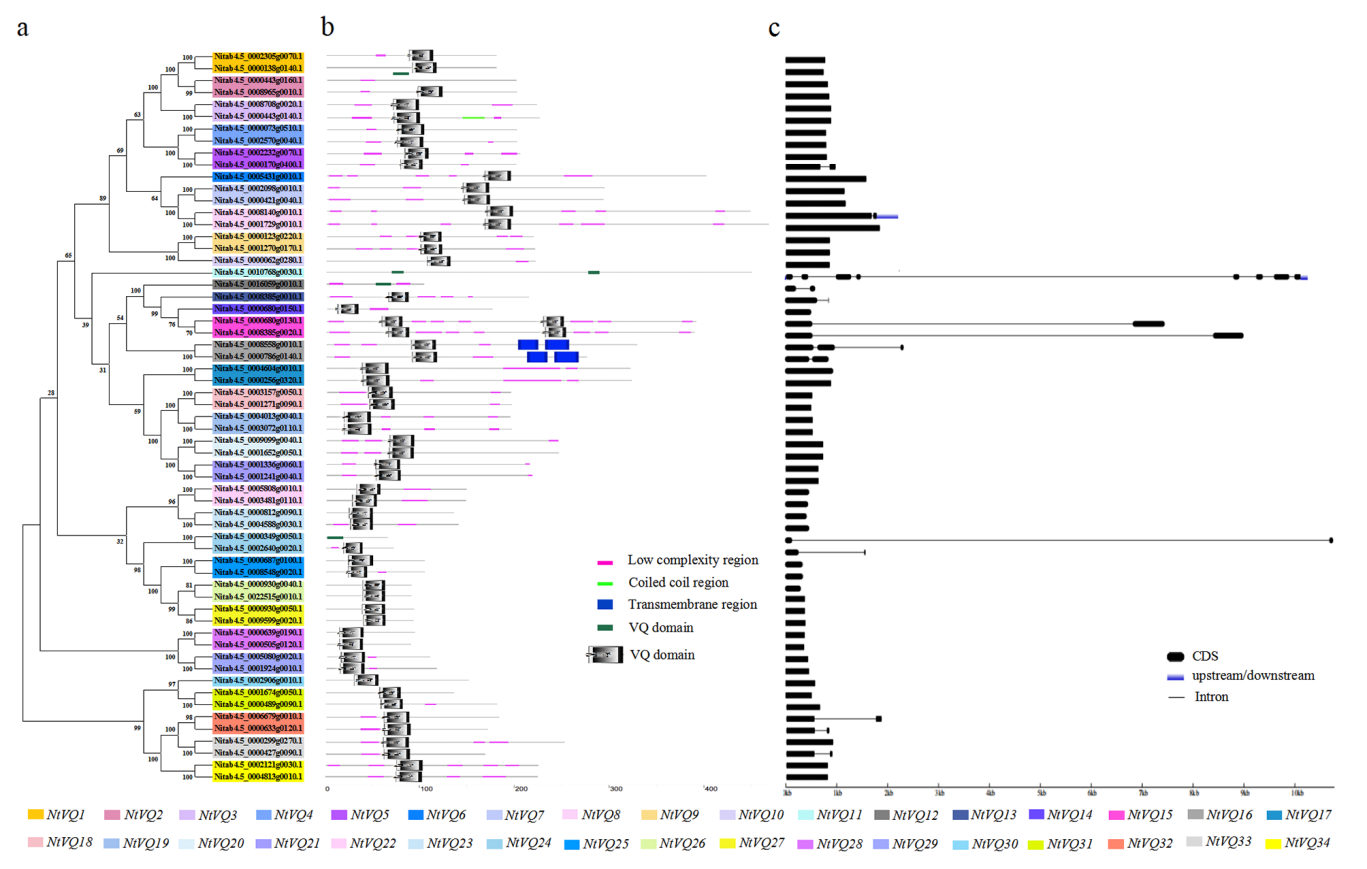
Genome wide organization of tobacco *NtVQ* genes a) Phylogenetic tree based on the protein sequences of sixty-one *NtVQ* genes. Phylogenetic tree was constructed using MEGA 7.0 by the neighbor-joining method with 1000 bootstrap. (b) Structures of NtVQ proteins: Conserved domains were showed in different colored boxes. (c) Exon-intron structure of *NtVQ* genes: black rectangles indicated coding sequence (CDS), blue indicated untranslated 5′- and 3′- regions, black lines indicated introns

A phylogenetic tree was constructed using sixty-one NtVQ protein sequences (Fig. 3a). The *NtVQ* genes could be divided into thirty-four categories (*NtVQ1* to *NtVQ34*) marked with different colours. The analysis of protein domain organization showed that all NtVQ proteins delineated the VQ-motif using SMART database and NCBI, and protein structures were highly similar (Fig. 3b). Protein structure of NtVQ3 (Nitab4.5_0000443g0140.1) contained a coiled-coil region. Protein structures of NtVQ16 (Nitab4.5_0008558g0010.1 and Nitab4.5_0000786g0140.1) had two transmembrane helix regions, respectively. The analysis of gene structure showed that *NtVQ* genes in the same branch shared a similar exon-intron distribution, except *NtVQ5*, *NtVQ8*, *NtVQ16* and *NtVQ33* (Fig. 3c). Fourteen *NtVQ* genes contained introns in the genomic sequences, the number of introns varied significantly from one to eight, indicating that *NtVQ* genes usually varied in the exon-intron distribution profile and gene length of the tobacco genomic sequences.

### Tissue expression pattern of *NtVQ* genes

**Fig. 4:**
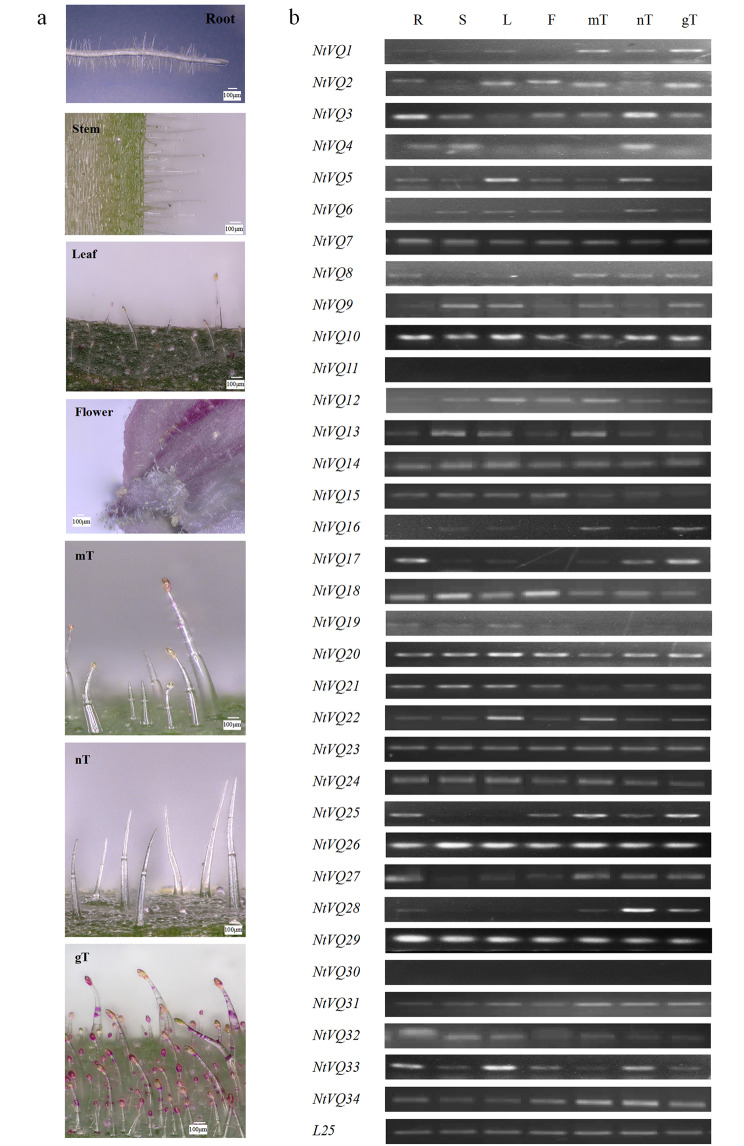
Expression patterns of thirty-four *NtVQ* genes in different tissues and trichomes (a) Epidermal phenotypes of tobacco roots, stems, leaves, flowers, mixed-trichome (mT), glandular-trichome (gT) and nonglandular-trichome (nT). (b) Gene expression profiles in different tissues and trichomes

To determine the potential functions of *NtVQ* genes in tobacco development, the expression profiles of thirty-four *NtVQ* genes were conducted in four tissues and three types of trichomes. Lots of trichomes were shown on the surface of stem and leaf, and tobacco root and flower were also covered with epidermis (Fig. 4a). As shown in Fig. 4b, the result of tissue expression profiles showed that *NtVQ11* and *NtVQ30* were barely expressed in different tissues including trichomes, *NtVQ16* were barely expressed in tissues, and *NtVQ19* were barely expressed in different type of trichomes. Seven genes (*NtVQ3*, *NtVQ8*, *NtVQ17*, *NtVQ25*, *NtVQ27*, *NtVQ29* and *NtVQ32*) were highly expressed in root, and *NtVQ28* was expressed only in root. Five genes (*NtVQ9*, *NtVQ13*, *NtVQ18*, *NtVQ21* and *NtVQ26*) were highly expressed in stem. Eight genes (*NtVQ2*, *NtVQ5*, *NtVQ10*, *NtVQ12*, *NtVQ20*, *NtVQ22*, *NtVQ24* and *NtVQ33*) were highly expressed in leaf. *NtVQ15* was highly expressed in flower, *NtVQ1* and *NtVQ8* were barely expressed in flower. These results forecasted that *NtVQ* genes played essential roles in tobacco tissues growth and development. Besides, eleven genes (*NtVQ8*, *NtVQ9*, *NtVQ12*, *NtVQ13*, *NtVQ16*, *NtVQ22*, *NtVQ24*, *NtVQ25*, *NtVQ26*, *NtVQ31* and *NtVQ34*) were highly expressed in mT. Four genes (*NtVQ1*, *NtVQ2*, *NtVQ17* and *NtVQ20*) were highly expressed in gT. Five genes (*NtVQ3*, *NtVQ4*, *NtVQ5*, *NtVQ28* and *NtVQ33*) were highly expressed in nT. The expression profiles in different type of trichomes firstly clarified the important functions of *NtVQ* genes in glandular- and nonglandular-trichome formation and development.

### Expression patterns of *NtVQ* genes following phytohormone treatments and abiotic stresses

**Fig. 5:**
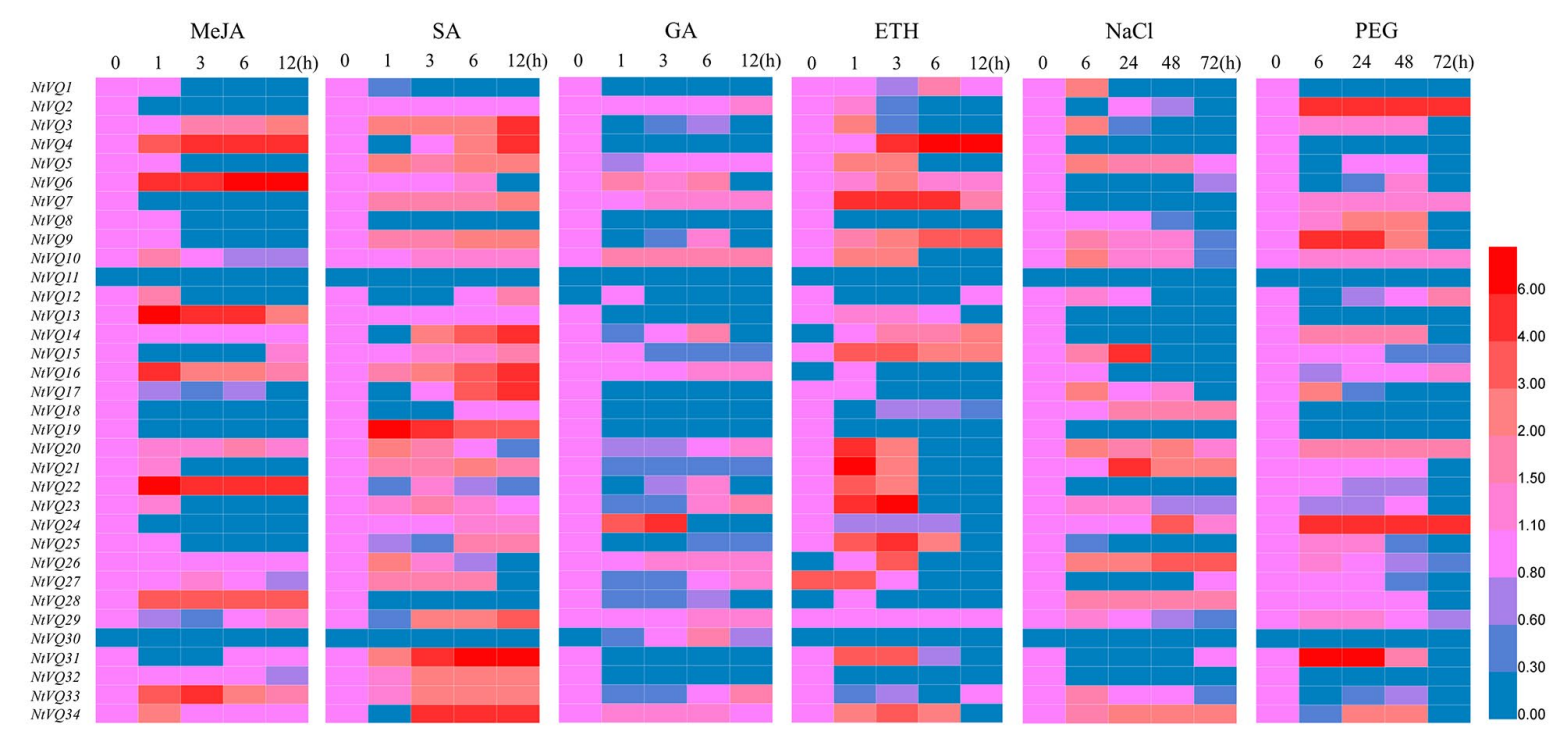
Expression profiles of thirty-four *NtVQ* genes under four phytohormones, NaCl and PEG stresses. The expression data from the semi-quantitative RT-PCR analysis were analyzed and visualized into heat maps using the TBtools software and MeV 4.8.1. The color scale represented relative expression levels with red and blue indicating increased or decreased transcript abundance, respectively

To elucidate the roles of *NtVQ* genes under different phytohormone, sqRT-PCR was conducted to achieve the relative expression values of each *NtVQ* gene (Fig. 5). The expression levels of thirteen genes (*NtVQ3*, *NtVQ4*, *NtVQ6*, *NtVQ13*, *NtVQ15*, *NtVQ16*, *NtVQ20*, *NtVQ22*, *NtVQ28*, *NtVQ29*, *NtVQ31*, *NtVQ33* and *NtVQ34*) increased following MeJA treatment. The expression levels of twenty-two genes (*NtVQ3*, *NtVQ4*, *NtVQ5*, *NtVQ7*, *NtVQ9*, *NtVQ10*, *NtVQ12*, *NtVQ14*, *NtVQ15*, *NtVQ16*, *NtVQ17*, *NtVQ18*, *NtVQ19*, *NtVQ21*, *NtVQ23*, *NtVQ24*, *NtVQ25*, *NtVQ29*, *NtVQ31*, *NtVQ32*, *NtVQ33* and *NtVQ34*) increased following SA treatment. The expression levels of eleven genes (*NtVQ2*, *NtVQ7*, *NtVQ10*, *NtVQ16*, *NtVQ20*, *NtVQ23*, *NtVQ26*, *NtVQ27*, *NtVQ29*, *NtVQ33* and *NtVQ34*) increased following GA treatment. The expression levels of eight genes (*NtVQ1*, *NtVQ4*, *NtVQ6*, *NtVQ7*, *NtVQ9*, *NtVQ14*, *NtVQ15* and *NtVQ29*) increased following ETH treatment. Among these genes, the expression levels of *NtVQ29* was simultaneously up-regulated following the four phytohormones treatments. These results uncovered that all *NtVQ* genes were involved in the intricate signaling pathways and each gene had different regulatory characteristics.

A systematic expression analysis of *NtVQ* genes was conducted following the abiotic stresses. The expression levels of eight genes (*NtVQ5*, *NtVQ18*, *NtVQ20*, *NtVQ21*, *NtVQ24*, *NtVQ26*, *NtVQ28* and *NtVQ34*) increased following high salinity stress. The expression levels of six genes (*NtVQ2*, *NtVQ7*, *NtVQ12*, *NtVQ16*, *NtVQ20* and *NtVQ24*) increased following PEG stress. *NtVQ20* and *NtVQ24* were simultaneously up-regulated following these two stresses. While the expression levels of fifteen genes (*NtVQ1*, *NtVQ3*, *NtVQ4*, *NtVQ6*, *NtVQ8*, *NtVQ13*, *NtVQ15*, *NtVQ17*, *NtVQ19*, *NtVQ22*, *NtVQ23*, *NtVQ25*, *NtVQ29*, *NtVQ32* and *NtVQ33*) were simultaneously down-regulated. These results predicated that most *NtVQ* genes showed the negative regulatory responses to abiotic stress.

### Transcriptional activity analysis of *NtVQ* genes

**Fig. 6:**
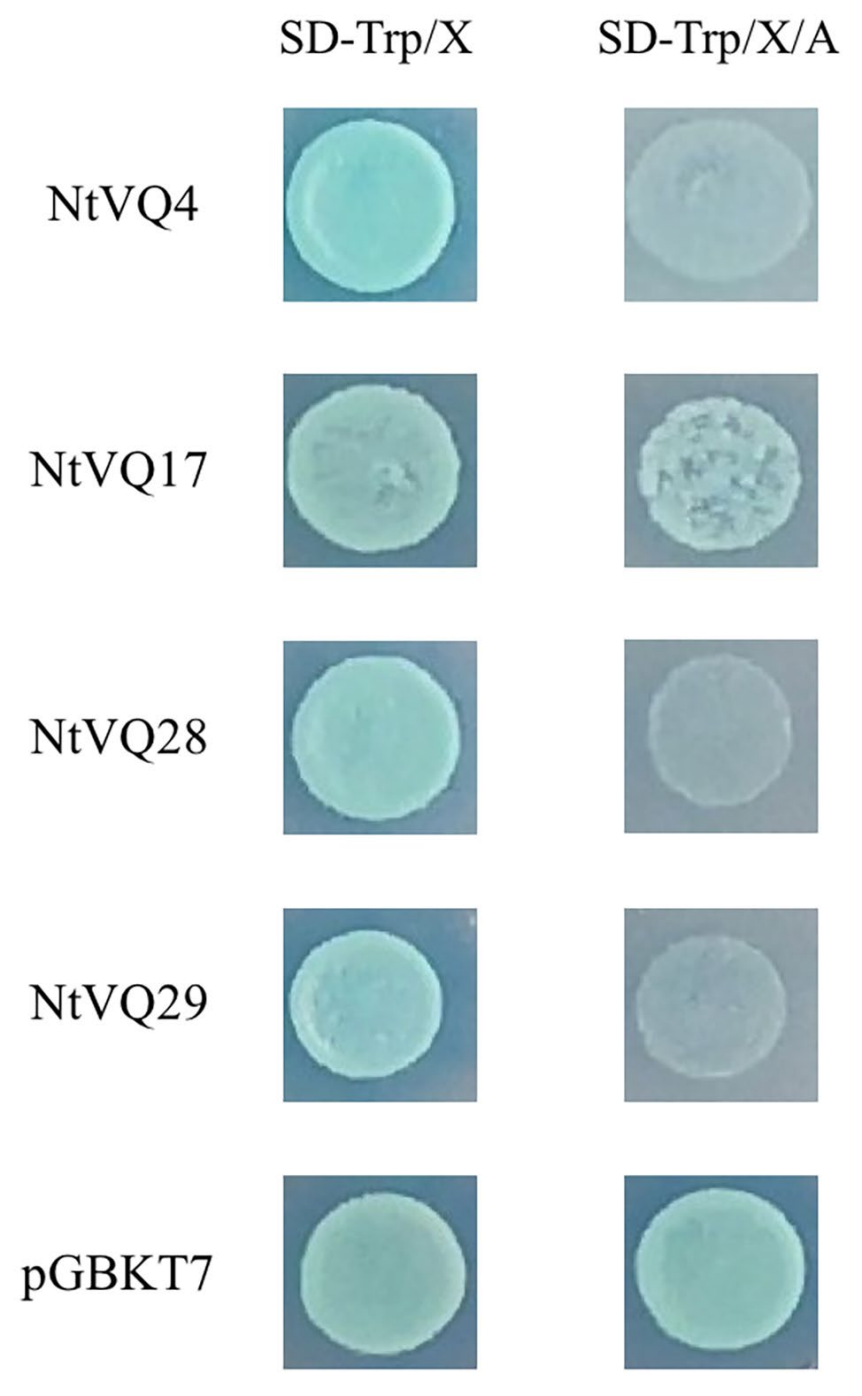
Transcriptional activity assay of several *NtVQ* genes. pGBKT7 was used as the control. The yeast colonies were cultivated and photographed after being cultured for 3 days at 30 °C

Several *NtVQ* genes have been chosen to investigate the potential transcriptional activity (Fig. 6). Compared with the control, pGBKT7-*NtVQ4*, pGBKT7-*NtVQ28* and pGBKT7-*NtVQ29* could only grow on SD/-Trp medium, while pGBKT7-*NtVQ17* grew normally on both selective media and showed alpha-galactosidase activity, which speculated that most *NtVQ* genes might have no transcription activity.

## Discussion

*VQ* gene has been extensively identified in various plants including angiosperms, gymnosperms and mosses. Some *VQ* gene were known to be related to the plant development and stress tolerance (Jiang et al. 2018; León et al. 2021). According to the tobacco genome data, sixty-one *NtVQ* genes containing VQ-motif were identified and their protein lengths ranged from 71 to 467 amino acids in the current study (Table 1), which was similar with these in *Arabidopsis* but smaller than these in moss (Li et al. 2014; Jing and Lin 2015). Then *NtVQ* gene structures, gene expression patterns, protein characteristics and their primary functions have already been confirmed.

### Gene duplication and phylogenetic analysis of *NtVQ* and *AtVQ* genes

Gene duplication event played a major role in plant genome rearrangement and expansion (Vision et al. 2000), and segregation duplication events were shown to provide references for the evolutionary relationship between *VQ* genes (Fig. 1), thereby enabling functional predictions (Panchy et al. 2016). For example, *AtVQ11* (*MVQ5*, AT1G80450), *AtVQ19* (*MVQ4*, AT3G15300) and *AtVQ33* (*MVQ3*, AT5G53830) interacted with specific subgroups of WRKY transcription factors and their proteins stability were mediated by phosphorylation (Pecher et al. 2014). The phylogenetic analysis revealed that the *VQ* genes from tobacco and *Arabidopsis* were classified into seven groups and the orthologous genes were clad in the same group (Fig. 2). Pecher (2014) has also proved that *AtVQ4*, *AtVQ6*, *AtVQ9*, *AtVQ13*, *AtVQ14*, *AtVQ31* and *AtVQ32* were all targeted by *MPK3* and *MPK6*, which speculated that tobacco NtVQ proteins from the group I and II might be involved in the phosphorylation process. These results indicated that the related VQ proteins in each group could be equipped with the similar functions.

### Structural and conserved domain analysis of *NtVQ* genes

Cultivated tobacco *N. tobacum* was an allotetraploid plant, thereby one gene might have two homologous sequences from two ancestors of *N. sylvestris* and *N. tomentosiformis* (Renny-Byfield et al. 2011). In this study, thirty-four *NtVQ* genes were grouped referring to the phylogenetic tree (Fig. 3a). Structure analysis showed that NtVQ proteins in the same group contained the similar type of motifs (Fig. 3b), indicating that close proteins shared similar functions. NtVQ3 contained a coiled-coil region which was found in more than two hundred proteins and used to predicate regions of protein discontinuity and folding stability (Lupas et al. 1991). NtVQ16 had two transmembrane helix regions and marked as the integral membrane protein (Krogh et al. 2001). Interestingly, most *VQ* genes in higher plants did not have any intron (Jing and Lin 2015), for instance, 90% *AtVQ* genes (Cheng et al. 2012) and 76% *PoVQ* genes (Chu et al. 2016) had no introns. Consistently, introns of twenty *NtVQ* genes were lost (Fig. 3c), which meant that *NtVQ* genes might have experienced different selective pressures during evolution.

### Analysis of *NtVQ* gene expression patterns in different tissues and trichomes

Tissue transcription patterns could exhibit genes involvement in functional or differential events. Thirty-four *AtVQ* genes were induced and differentially expressed in different tissues (Cheng et al. 2012). Herein, *NtVQ11* and *NtVQ30* were barely expressed in different tissues including trichomes. Consistently, the homologous gene *AtVQ20* expressed strongly in the male gametophytic tissues, but barely in seedling, leaf, stem and root (Lei et al. 2017). *AtVQ12* was mainly expressed in the root and leaf (Wang et al. 2015). *AtVQ29* was mainly expressed in the root, leaf, hypocotyl and silique base (Jing and Lin 2015). Moreover, *GmVQ58* was highest expressed in the cotton leaf and root (Li et al. 2020). Almost all *CsVQ* genes were more highly expressed in root, stem and leaf of tea plant, while only a few genes were more highly expressed in the flower (Guo et al. 2018). Some uncharacterized VQ proteins were also found in response to meditate plant growth. In our results, the tissues expression profiles showed that almost all *NtVQ* genes were responsive to organogenesis, thus pointing to the important regulatory roles of *NtVQ* genes in tobacco development.

Trichomes were one of the most important accommodative traits in plants (Ishida et al. 2008). Trichomes were associated with several important features that were involved in phytohormone responses, resistance to biotic and abiotic stress (Schuurink and Tissier 2020; Yan et al. 2021). gT and nT not only formed the physical barriers against UV radiation, waster loss and excess light (Schuurink and Tissier 2020), but also were crucial in the process of chemical secretion such as phenylpropanoids, alkaloids, sugars and some other metabolic compounds against insects and arthropods (Yang and Ye 2013; Maurya et al. 2019). However, the expression patterns and molecular regulatory mechanisms of *NtVQ* genes in trichomes had never been published. Here, eleven *NtVQ* genes highly expressed in mT from K326, four *NtVQ* genes highly expressed in gT from T.I.1068, and five *NtVQ* genes highly expressed in nT from T.I.1112, which made it clear that *NtVQ* genes had unique potentials and momentous values to participate in trichomes development responsive to the multiple external stimulus.

### Gene expression patterns under environmental stresses

Plants had generated kinds of effective defense systems against biotic and abiotic stresses. Jasmonic acid (JA) and SA were two of the best-known signaling molecules that regulated plant development and triggered defense responses (Dubrovsky 2005; Jing and Lin 2015) pointed that *VQ* genes were involved in JA- and SA-meditated defense responses.

In this study, *NtVQ4* was significantly and consistently up-regulated upon JA treatment (Fig. 5), and its highly homologous gene *AtVQ22* (*JAV1*) transcript significantly increased and negatively regulated the transcriptional activity of *WRKY28*/*51* involved in the JA-meditated defense response (Andreasson et al. 2005; Hu et al. 2013a; Yan et al. 2018). Moreover, *OsVQ13* positively regulated JA-meditated grain size by activating the OsMPK6-OsWRKY45 signaling pathway in rice (Uji et al. 2019). The expression levels of *MaVQ5* was up-regulated after MeJA treatment (Ye et al. 2016). Moreover, the roles of JA in promoting leaf senescence and epidermogenesis were affirmed in many plant species (Zhang et al. 2019; Wang et al. 2022). *AtVQ18*, *AtVQ26* and *ZmVQ52* could effectively regulate JA-mediated leaf senescence (Pan et al. 2018; Yu et al. 2019). Herein, a detailed expression analysis revealed that nineteen *NtVQ* genes were obviously involved in the formation and development of gT and nT (Fig. 4), and thirteen *NtVQ* genes were up-regulated after MeJA treatment (Fig. 5). These results proved that *NtVQ* genes emitted important response functions to JA signaling, which was similar with the result of around 32% *AtVQ* genes up-regulated in senescing leaves (Schmid et al. 2005).

*NtVQ34* were highly expressed after SA treatment (Fig. 5), and its homologous gene *AtVQ21* played a positive role in SA signaling (Andreasson et al. 2005). While *NtVQ22* and *NtVQ23* were not sensitive to SA, which was opposite to the functions of their highly homologous genes *AtVQ16* (*SIB2*) and *AtVQ23* (*SIB1*) strongly induced by SA treatment (Xie et al. 2010; Lai et al. 2011). These results verified that *NtVQ* genes interlaced to form a variety of complex regulatory mechanisms in tobacco. In addition, most *NtVQ* genes were up-regulated after SA treatment and conjectured to play the important roles in SA-meditated plant growth, development and resistance responses, which was consistent with the results that thirty-four *AtVQ* genes were responsive to SA treatment (Cheng et al. 2012), and sixteen *VvVQ* genes were induced by SA treatment (Wang et al. 2015).

Recent studies in *Arabidopsis* and other crop species highlighted the emerging key roles for GA and ETH in the regulation of nearly all aspects of plant organs growth and yield under abiotic stress (Yamaguchi 2008; Dubois et al. 2018). In this study, part of *NtVQ* genes were positively induced after these two phytohormone treatments. The *VQ* genes from other plant also showed the similar function, for example, *PbrVQ9* was the top highly expressed gene after GA treatment (Cao et al. 2018), *VvVQ2* was highly up-regulated in grapevine after ETH treatment (Wang et al. 2015).

Salt and drought were the main factors of reducing crop production. The expression levels of *NtVQ16* and *NtVQ17* decreased after NaCl treatment (Fig. 5), while their homologous gene *AtVQ9* transcript increased (Perruc et al. 2004; Hu et al. 2013b). Some studies have indicated that some up-regulated *VQ* genes might have a negative effect on abiotic stress resistance. *AtVQ9* expression was induced by salinity stress, but *AtVQ9* overexpression increased plants hypersensitive to salinity stress (Hu et al. 2013b), which conjectured that the functional roles of *NtVQ* genes to abiotic stresses were characterized by inconsistency. Moreover, *AtVQ15* (*AtCaMBP25*) was induced by various abiotic stresses, including dehydration and high salinity, and *AtVQ15* overexpression plants displayed increasing sensitivity to both NaCl and mannitol (Perruc et al. 2004; Hu et al. 2013b). *AtVQ24*, a closed protein of *AtVQ15*, was clarified to have the negative role in osmotic stress (Hu et al. 2013b). And their homologous tobacco genes *NtVQ9* and *NtVQ10* showed the similar negative regulation. Besides, *GhVQ18* and *GhVQ84* were highly expressed under NaCl and PEG treatments (Chen et al. 2020b). Bamboo *PeVQ28* overexpression in *Arabidopsis* showed increased resistance to salinity stress (Cheng et al. 2020). Here, eight and six *NtVQ* genes were up-regulated after NaCl and PEG treatments, respectively. While the expression profiles of most *NtVQ* genes decreased. Previous studies showed the consistent results that ten *BrVQ* genes (Zhang et al. 2015), eight *PtVQ* genes (Chu et al. 2016), eighteen *VvVQ* genes (Wang et al. 2015), and twenty-two *OsVQ* genes (Kim et al. 2013) were up-regulated after drought stress. And most *VQ* genes from poplar (Chu et al. 2016), maize (Song et al. 2016), and tea (Guo et al. 2018) were also responsive to drought or PEG and NaCl stress.

### Most *NtVQ* genes lacked the transcriptional activity

Studies have proved that *AtVQ1* and *AtVQ10* did not have transcriptional activity (Jing and Lin 2015). Consistently, the homologous genes *NtVQ28* and *NtVQ29* were shown no self-activating activity (Fig. 6), and *NtVQ4* also showed no self-activating activity in the Y2H assay. Acted as the homologous gene of *AtVQ14* (IKU1/MVQ9), *NtVQ17* displayed the transcriptional activity, which was same with the result of the most prominent interactions between MPK3, MPK6 and MVQ proteins from group I and II (Pecher et al. 2014). All these data predicted that most *NtVQ* genes acted as transcription regulators might have no transcriptional activity, and functions of *NtVQ* genes, especially *NtVQ17*, needed further verification and evaluation.

## Conclusions

The transcriptions of *VQ* genes were modulated by multiple endogenous and environmental signals, consistent with their diverse roles in stress responses, plant growth and development. Study about *NtVQ* gene family provided a glimpse into the potential biological functions in tissues development, trichomes formation, and resistance to abiotic stress, indicating that members of *NtVQ* family played important roles in plant growth and responses to environmental conditions.

## Electronic supplementary material

Below is the link to the electronic supplementary material.

Supplementary Material 1

Supplementary Material 2

## Data Availability

All data analyzed during this study are included in this published article and its supplementary information files.
