# Supplementary material for: Characterization and the comprehensive expression analysis of tobacco valine-glutamine genes in response to trichomes development and stress tolerance
Source: Bot Stud. 2023 Jul 10;64:18. doi: 10.1186/s40529-023-00376-x (PMC10329969; doi:10.1186/s40529-023-00376-x)
Supplement: Supplementary file 1 — Supplementary Material 1 [file 40529_2023_376_MOESM1_ESM.docx]

Dear editor,

Thank you for your time and effort in handling our manuscript entitled **“*Characterization and the comprehensive expression analysis of tobacco valine-glutamine genes in response to trichomes development and stress tolerance*”**. We have addressed all the concerns raised by the reviewer with the detailed responses with the comments point-by-point, and re-examined the sentences and words marked in red in the revised manuscript (line 46, 91, 98-101, 110, 129, 137, 129, 208 and 345). Now we trust that the manuscript is now acceptable for publication in ***Botanical Studies***.

Thank you again for your time and effort.

Sincerely yours,

Hong Cui

Henan Agricultural University

Zhengzhou, 450002, China

E-mail: cuihong@henau.edu.cn
