# Supplementary material for: Characterization and the comprehensive expression analysis of tobacco valine-glutamine genes in response to trichomes development and stress tolerance
Source: Bot Stud. 2023 Jul 10;64:18. doi: 10.1186/s40529-023-00376-x (PMC10329969; doi:10.1186/s40529-023-00376-x)
Supplement: Supplementary file 2 — Supplementary Material 2 [file 40529_2023_376_MOESM2_ESM.docx]

Dear Reviewer,

Thank you for your time and effort in handling our manuscript. We have addressed all the concerns raised by the reviewer with the revision and discussion, and we trust that the manuscript is now acceptable for publication in ***Botanical Studies***. Below please find a detailed response with the comments point-by-point. Comments are pasted below in italics and our response are in regular and blue text.

Thank you again for your time and effort.

Sincerely yours,

Hong Cui

Henan Agricultural University

Zhengzhou, 450002, China

E-mail: cuihong@henau.edu.cn

*1. Lines 93-106: "Identification of tobacco VQ genes". More detail method should be added. Please refer to Guo et al (2014 J Exp Bot, 65: 6, 1513) and Zhang et al (2021 BMC Genomics, 22, 1: 221).*

Response: We thank the reviewer for pointing this out.

We have added some more detailed methods about “Identification of tobacco VQ genes” in line 96-101 in the revised manuscript. Besides, we have supplemented three corresponding literatures in Reference.

*2. Lines 106, 132: The relevant literature should be added.*

Response: We thank the reviewer for pointing these out.

We have added the relevant literatures in line 110 and 137 in the revised manuscript and Reference.

*3. Line 225: Please clarify the choice of these VQ genes to be analyzed by transcriptional activity. Or elsewhere, in the discussion probably. Please give an explanation on the reasons why VQ4/28/29 possess no activation ability in yeast (maybe it needs a co-factor? it acts in a molecular complex?)*

Response: We thank the reviewer for pointing these out.

*NtVQ4* was significantly and consistently up-regulated upon JA treatment (Figure 5). Its highly homologous gene *AtVQ22* (Jasmonate-associated VQ motif gene1, *JAV1*) showed no antoactivation feature and was verified to negatively regulate the transcriptional activity of *WRKY28*/*51* involved in the JA-meditated defense response (Andreasson et al. 2005; Hu et al. 2013a; Yan et al. 2018). *NtVQ4* will be the focus to study the mechanism of growth and development meditated by JA in our future study, along with the published research of our research group, such as JA-meditated trichome formation (Yan et al. 2022; Zhang et al. 2023), and JA-meditated plant disease resistance (Zhang et al. 2019). So we analyzed *NtVQ4* transcriptional activity.

Some studies have proved that *AtVQ1* and *AtVQ10* did not have transcriptional activity (Jing and Lin 2015). Then their homologous genes *NtVQ28* and *NtVQ29* were used to complete the transcriptional activity analysis, which could verify the similarity of functional characteristics of homologous genes. Besides, the expression levels of *NtVQ29* was simultaneously up-regulated following the four phytohormones treatments (Figure 5). Then, both of *NtVQ28* and *NtVQ29* were carried for transcriptional activity analysis.

Similarly, *NtVQ17* was the homologous genes of *AtVQ14*. *AtVQ14* (HAIKU1, *IKU1*, or *MVQ9*) regulated endosperm growth and seeds size by interacting with *AtWRKY10* (Wang et al. 2010), and was targeted by *MPK3* and *MPK6*, which speculated that tobacco *NtVQ* proteins from the group I and II might be involved in the phosphorylation process and acted like *MVQs* (Pecher et al. 2014). Therefore, we chose *NtVQ17* for further analysis, which was same with the result of the most prominent interactions between *MPK3*, *MPK6* and *MVQ* proteins from group I and II (Pecher et al. 2014).

Andreasson E, Jenkins T, Brodersen P, Thorgrimsen S, Petersen NH, Zhu S, Qiu JL, Micheelsen P, Rocher A, Petersen M, Newman MA, Bjorn Nielsen H, Hirt H, Somssich I, Mattsson O, Mundy J (2005) The MAP kinase substrate *MKS1* is a regulator of plant defense responses. EMBO J. 24: 2579-2589. doi: 10.1038/sj.emboj.7600737.

Hu P, Zhou W, Cheng Z, Fan M, Wang L, Xie D (2013a). *JAV1* controls jasmonate-regulated plant defense. Mol Cell. 50: 504-515. doi: 10.1016/j.molcel.2013.04.027.

Yan C, Fan M, Yang M, Zhao J, Zhang W, Su Y, Xiao L, Deng H, Xie D (2018) Injury activates Ca^2+^/calmodulin-dependent phosphorylation of JAV1-JAZ8-WRKY51 complex for jasmonate biosynthesis. Mol Cell. 70: 136-149. e7. doi: 10.1016/j.molcel.2018.03.013.

Yan X, Cui L, Liu X, Cui Y, Wang Z, Zhang H, Chen L, Hong Cui (2022) *NbJAZ3* is required for jasmonate-meditated glandular trichome development in *Nicotiana benthamiana*. Physiol Plantarum. 174: e13666. doi: 10.1111/ppl.13666.

Zhang G, Yan X, Zhang S, Zhu Y, Zhang X, Qiao H, van Nocker S, Li Z, Wang X (2019) The jasmonate-ZIM domain gene *VqJAZ4* from the Chinese wild grape *Vitis quinquangularis* improves resistance to powdery mildew in *Arabidopsis thaliana*. Plant Physiol Biochem. 143: 329-339. doi: 10.1016/j.plaphy.2019.09.018.

Zhang H, Xu H, Xu M, Yan X, Wang Z, Yu J, Lei B, Cui H (2023) Transcription factors *NtHD9* and *NtHD12* control long glandular trichome formation via jasmonate signaling, Plant Physiol. kiad003. doi.org/10.1093/plphys/kiad003.

Jing Y, Lin R (2015) The VQ motif-containing protein family of plant-specific transcriptional regulators. Plant Physiol. 169: 371-378. doi: 10.1104/pp.15.00788.

Wang A, Garcia D, Zhang H, Feng K, Chaudhury A, Berger F, Peacock WJ, Dennis ES, Luo M (2010) The VQ motif protein IKU1 regulates endosperm growth and seed size in *Arabidopsis*. Plant J. 63: 670-679. doi: 10.1111/j.1365-313X.2010.04271.x.

Pecher P, Eschen-Lippold L, Herklotz S, Kuhle K, Naumann K, Bethke G, Uhrig J, Weyhe M, Scheel D, Lee J (2014) The *Arabidopsis thaliana* mitogen-activated protein kinases MPK3 and MPK6 target a subclass of 'VQ-motif'-containing proteins to regulate immune responses. New Phytol. 203: 592-606. doi: 10.1111/nph.12817.

*4. The reviewer cannot see the labels in Figs. The high definition of submitted images must be at least 300 dpi.*

Response: We thank the reviewer for pointing these out.

We have recomposed all the figures in the revised manuscript.

*5. The English writing is obscure which needs to be further revised by the professorial editing serves or by native speakers.*

Response: We thank the reviewer for pointing these out.

Before submitting, we have invited the professor Dr. Hongying Zhang who publishes the high-level paper to revise the sentences (Zhang et al. 2023, Plant Physiol,kiad003. doi.org/10.1093/plphys/kiad003). After your suggestion, we have invited the professor Dr. Golam Jalal Ahammed who is an Associate Professor at Henan University of Science and Technology to revise the language again, thus hoping to improve the reading fluency.
